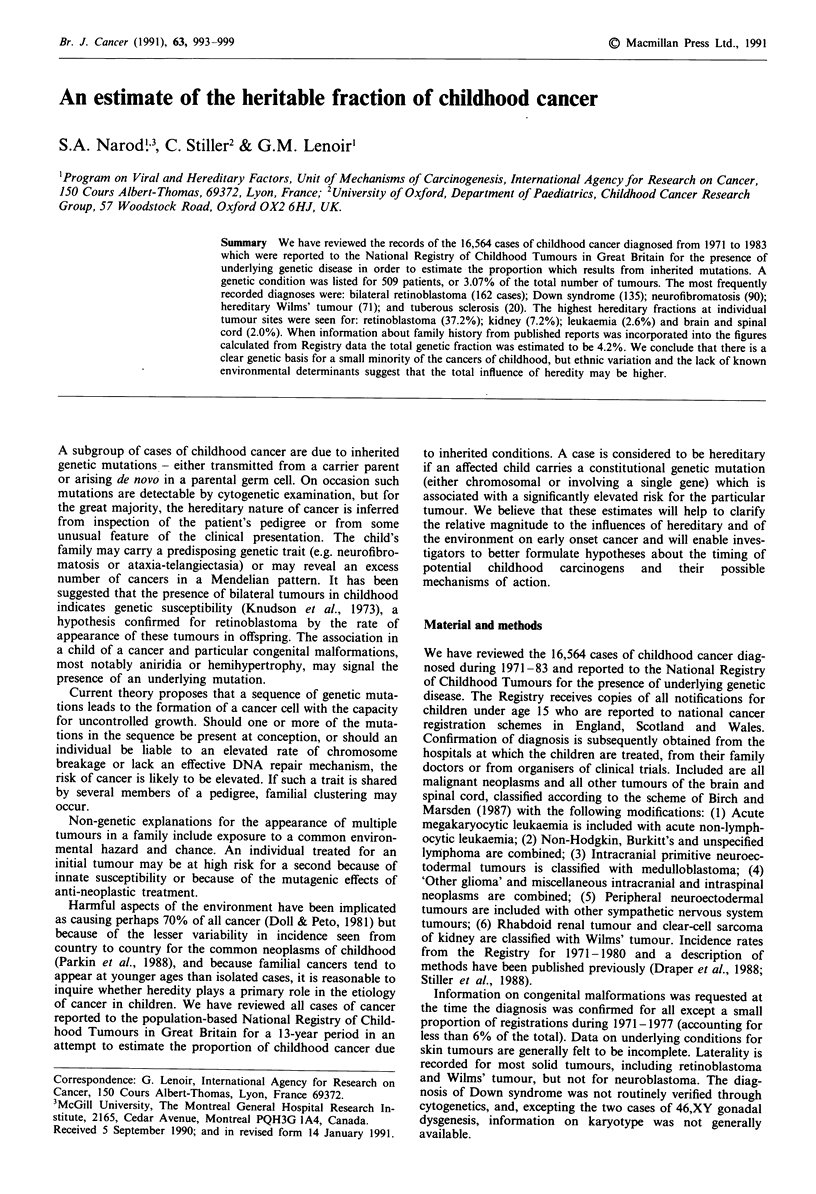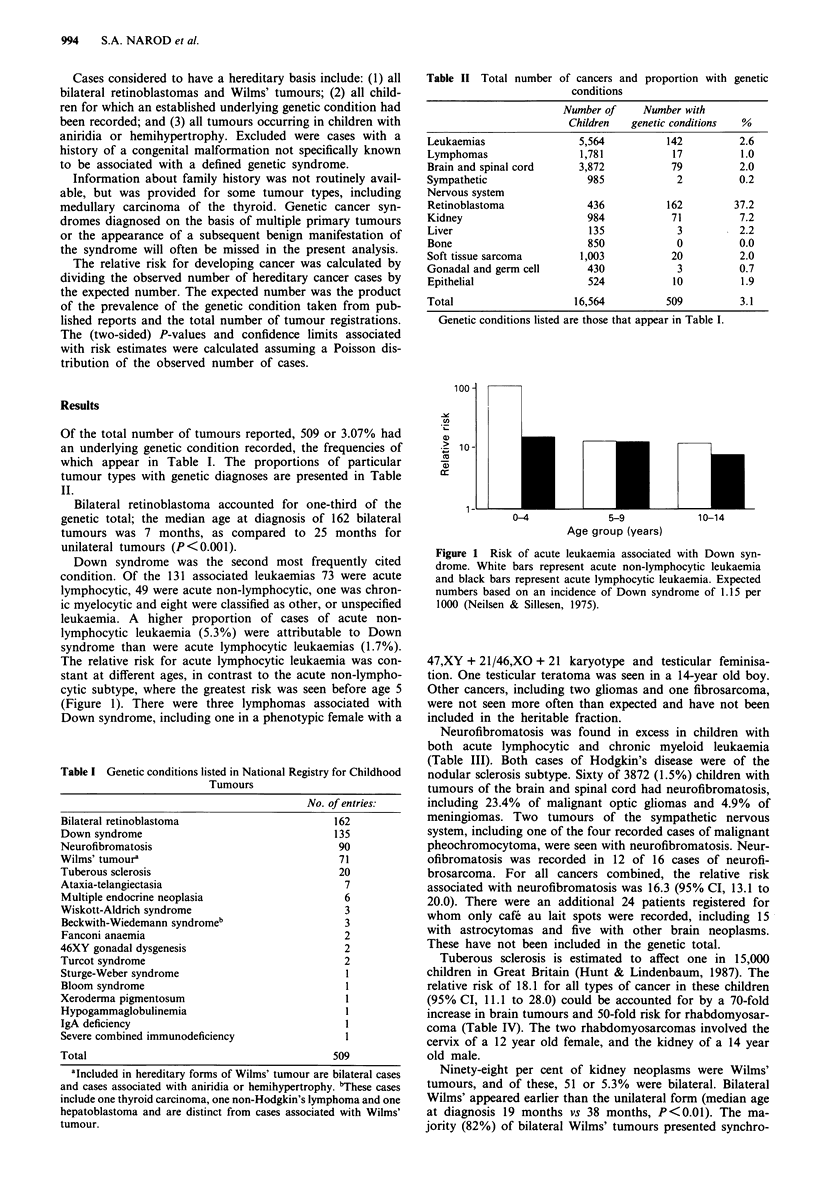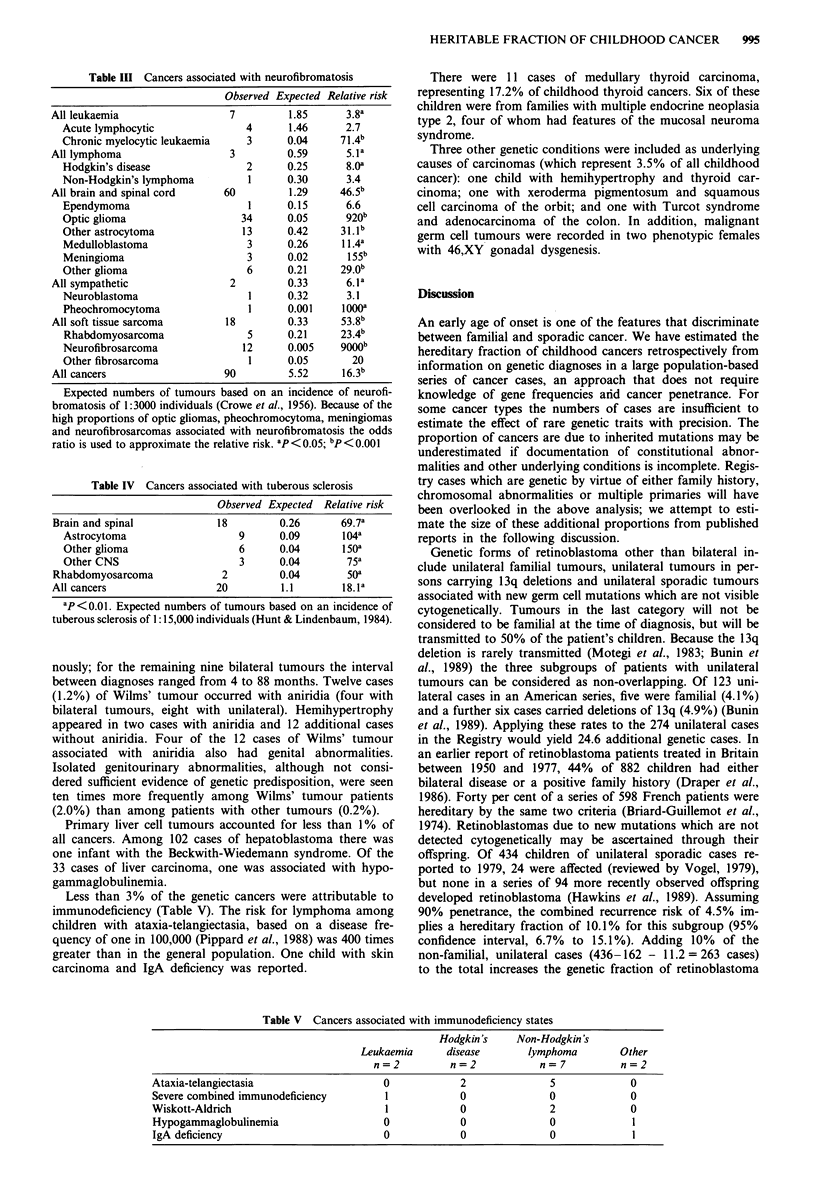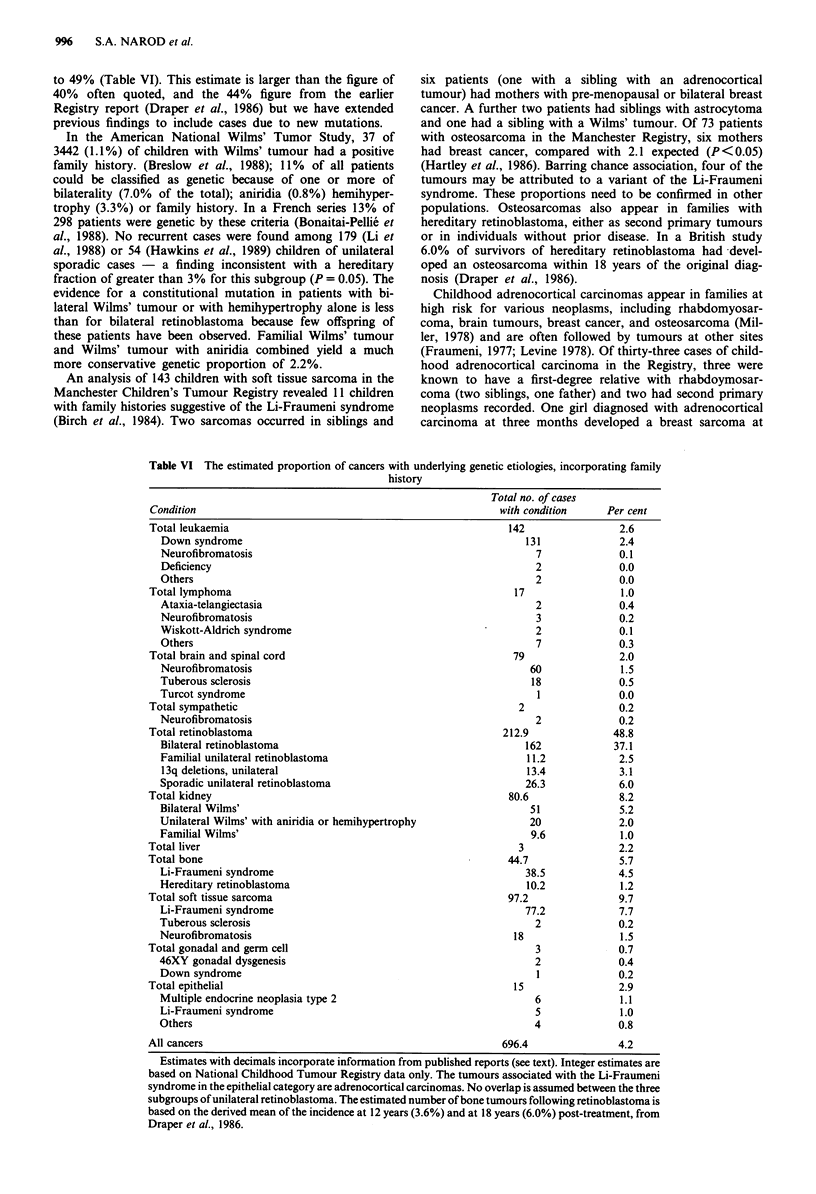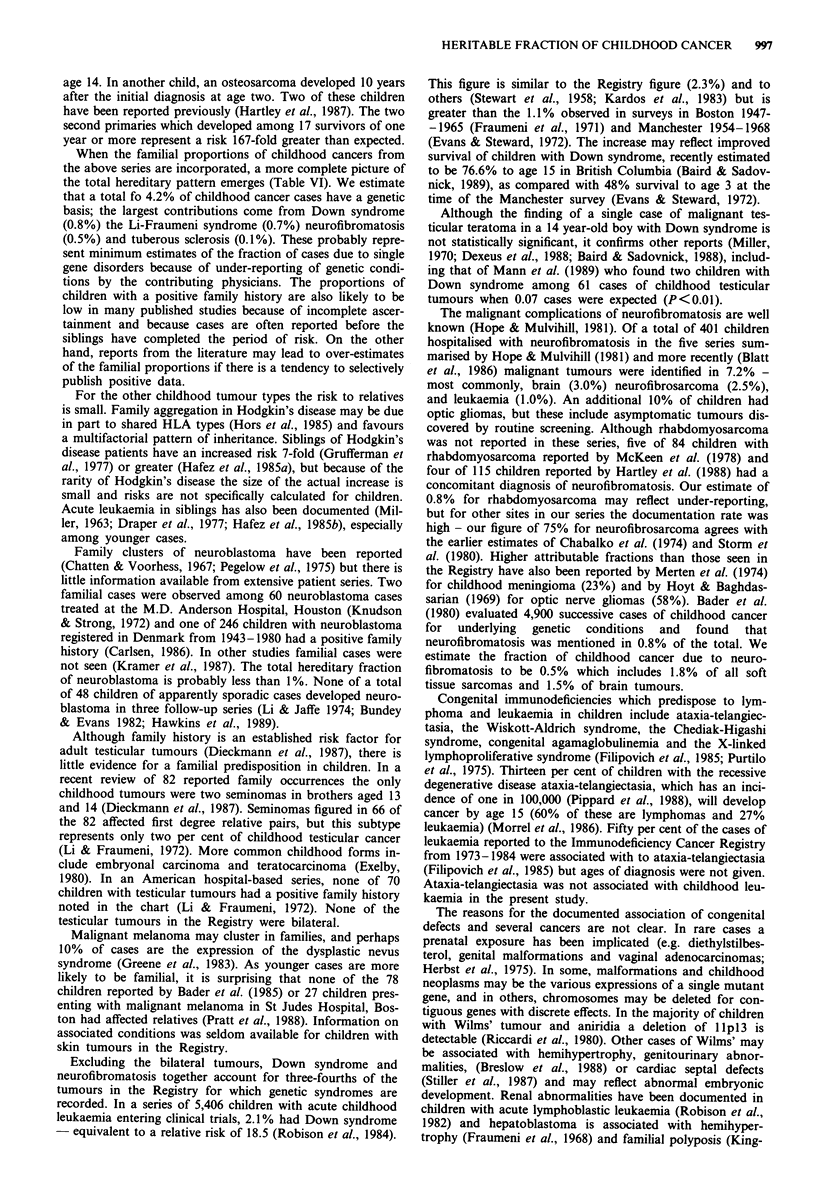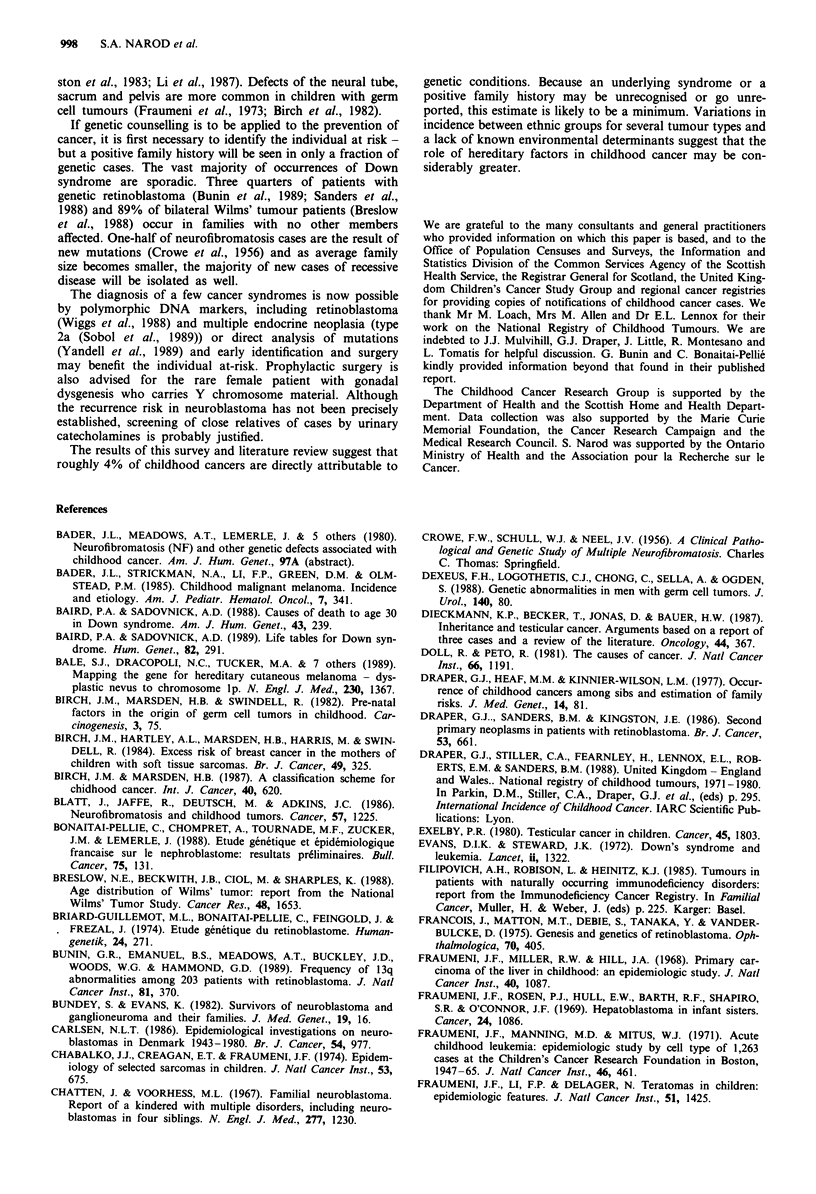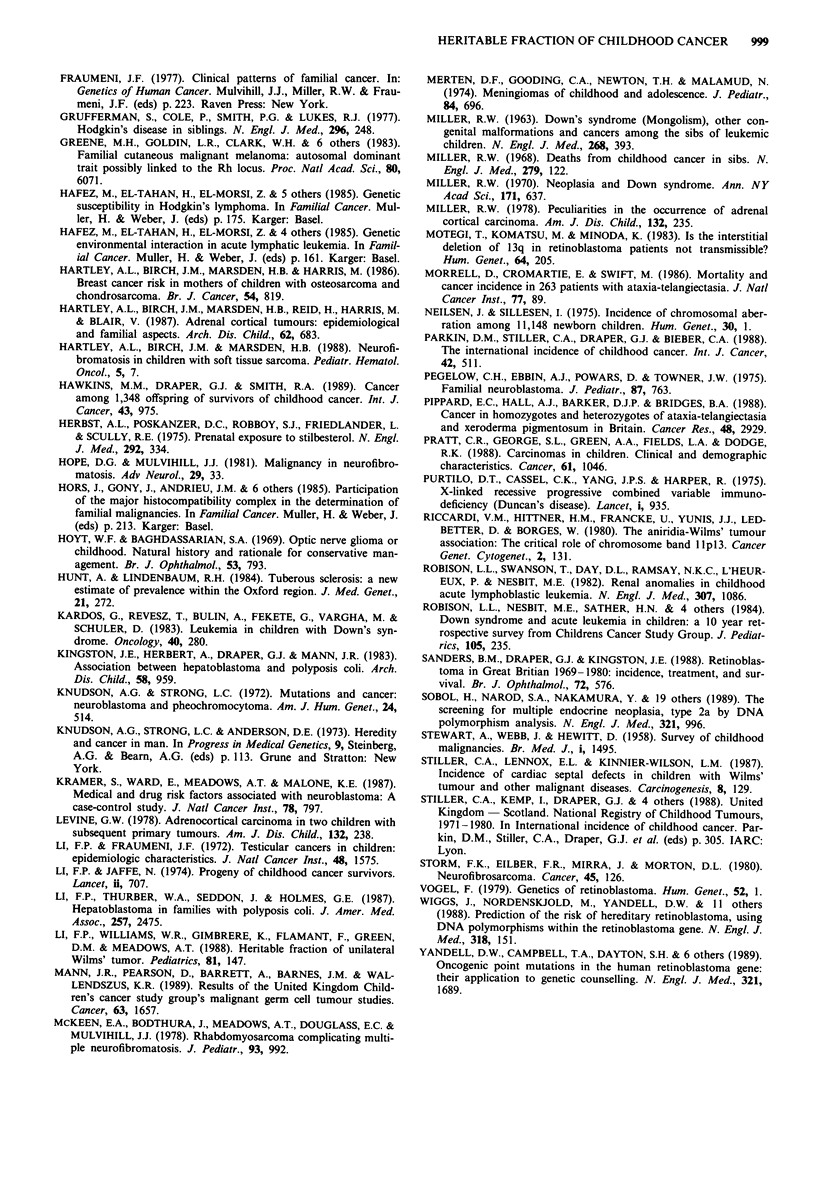# An estimate of the heritable fraction of childhood cancer.

**DOI:** 10.1038/bjc.1991.216

**Published:** 1991-06

**Authors:** S. A. Narod, C. Stiller, G. M. Lenoir

**Affiliations:** Program on Viral and Hereditary Factors, International Agency for Research on Cancer, Lyon, France.

## Abstract

We have reviewed the records of the 16,564 cases of childhood cancer diagnosed from 1971 to 1983 which were reported to the National Registry of Childhood Tumours in Great Britain for the presence of underlying genetic disease in order to estimate the proportion which results from inherited mutations. A genetic condition was listed for 509 patients, or 3.07% of the total number of tumours. The most frequently recorded diagnoses were: bilateral retinoblastoma (162 cases); Down syndrome (135); neurofibromatosis (90); hereditary Wilms' tumour (71); and tuberous sclerosis (20). The highest hereditary fractions at individual tumour sites were seen for: retinoblastoma (37.2%); kidney (7.2%); leukaemia (2.6%) and brain and spinal cord (2.0%). When information about family history from published reports was incorporated into the figures calculated from Registry data the total genetic fraction was estimated to be 4.2%. We conclude that there is a clear genetic basis for a small minority of the cancers of childhood, but ethnic variation and the lack of known environmental determinants suggest that the total influence of heredity may be higher.


Br. J. Cancer (1991), 63, 993-999 © Macmillan Press Ltd., 1991

### An estimate of the heritable fraction of childhood cancer

S.A. Narod[1,3], C. Stiller[2] & G.M. Lenoir[1]

[1]*Program on Viral and Hereditary Factors, Unit of Mechanisms of Carcinogenesis, International Agency for Research on Cancer, 150 Cours Albert-Thomas, 69372, Lyon, France;* [2]*University of Oxford, Department of Paediatrics, Childhood Cancer Research Group, 57 Woodstock Road, Oxford OX2 6HJ, UK.*

**Summary** We have reviewed the records of the 16,564 cases of childhood cancer diagnosed from 1971 to 1983 which were reported to the National Registry of Childhood Tumours in Great Britain for the presence of underlying genetic disease in order to estimate the proportion which results from inherited mutations. A genetic condition was listed for 509 patients, or 3.07% of the total number of tumours. The most frequently recorded diagnoses were: bilateral retinoblastoma (162 cases); Down syndrome (135); neurofibromatosis (90); hereditary Wilms' tumour (71); and tuberous sclerosis (20). The highest hereditary fractions at individual tumour sites were seen for: retinoblastoma (37.2%); kidney (7.2%); leukaemia (2.6%) and brain and spinal cord (2.0%). When information about family history from published reports was incorporated into the figures calculated from Registry data the total genetic fraction was estimated to be 4.2%. We conclude that there is a clear genetic basis for a small minority of the cancers of childhood, but ethnic variation and the lack of known environmental determinants suggest that the total influence of heredity may be higher.

A subgroup of cases of childhood cancer are due to inherited genetic mutations – either transmitted from a carrier parent or arising *de novo* in a parental germ cell. On occasion such mutations are detectable by cytogenetic examination, but for the great majority, the hereditary nature of cancer is inferred from inspection of the patient's pedigree or from some unusual feature of the clinical presentation. The child's family may carry a predisposing genetic trait (e.g. neurofibromatosis or ataxia-telangiectasia) or may reveal an excess number of cancers in a Mendelian pattern. It has been suggested that the presence of bilateral tumours in childhood indicates genetic susceptibility (Knudson *et al.*, 1973), a hypothesis confirmed for retinoblastoma by the rate of appearance of these tumours in offspring. The association in a child of a cancer and particular congenital malformations, most notably aniridia or hemihypertrophy, may signal the presence of an underlying mutation.

Current theory proposes that a sequence of genetic mutations leads to the formation of a cancer cell with the capacity for uncontrolled growth. Should one or more of the mutations in the sequence be present at conception, or should an individual be liable to an elevated rate of chromosome breakage or lack an effective DNA repair mechanism, the risk of cancer is likely to be elevated. If such a trait is shared by several members of a pedigree, familial clustering may occur.

Non-genetic explanations for the appearance of multiple tumours in a family include exposure to a common environmental hazard and chance. An individual treated for an initial tumour may be at high risk for a second because of innate susceptibility or because of the mutagenic effects of anti-neoplastic treatment.

Harmful aspects of the environment have been implicated as causing perhaps 70% of all cancer (Doll & Peto, 1981) but because of the lesser variability in incidence seen from country to country for the common neoplasms of childhood (Parkin *et al.*, 1988), and because familial cancers tend to appear at younger ages than isolated cases, it is reasonable to inquire whether heredity plays a primary role in the etiology of cancer in children. We have reviewed all cases of cancer reported to the population-based National Registry of Childhood Tumours in Great Britain for a 13-year period in an attempt to estimate the proportion of childhood cancer due to inherited conditions. A case is considered to be hereditary if an affected child carries a constitutional genetic mutation (either chromosomal or involving a single gene) which is associated with a significantly elevated risk for the particular tumour. We believe that these estimates will help to clarify the relative magnitude to the influences of hereditary and of the environment on early onset cancer and will enable investigators to better formulate hypotheses about the timing of potential childhood carcinogens and their possible mechanisms of action.

### Material and methods

We have reviewed the 16,564 cases of childhood cancer diagnosed during 1971–83 and reported to the National Registry of Childhood Tumours for the presence of underlying genetic disease. The Registry receives copies of all notifications for children under age 15 who are reported to national cancer registration schemes in England, Scotland and Wales. Confirmation of diagnosis is subsequently obtained from the hospitals at which the children are treated, from their family doctors or from organisers of clinical trials. Included are all malignant neoplasms and all other tumours of the brain and spinal cord, classified according to the scheme of Birch and Marsden (1987) with the following modifications: (1) Acute megakaryocytic leukaemia is included with acute non-lymphocytic leukaemia; (2) Non-Hodgkin, Burkitt's and unspecified lymphoma are combined; (3) Intracranial primitive neuroectodermal tumours is classified with medulloblastoma; (4) 'Other glioma' and miscellaneous intracranial and intraspinal neoplasms are combined; (5) Peripheral neuroectodermal tumours are included with other sympathetic nervous system tumours; (6) Rhabdoid renal tumour and clear-cell sarcoma of kidney are classified with Wilms' tumour. Incidence rates from the Registry for 1971–1980 and a description of methods have been published previously (Draper *et al.*, 1988; Stiller *et al.*, 1988).

Information on congenital malformations was requested at the time the diagnosis was confirmed for all except a small proportion of registrations during 1971–1977 (accounting for less than 6% of the total). Data on underlying conditions for skin tumours are generally felt to be incomplete. Laterality is recorded for most solid tumours, including retinoblastoma and Wilms' tumour, but not for neuroblastoma. The diagnosis of Down syndrome was not routinely verified through cytogenetics, and, excepting the two cases of 46,XY gonadal dysgenesis, information on karyotype was not generally available.

Correspondence: G. Lenoir, International Agency for Research on Cancer, 150 Cours Albert-Thomas, Lyon, France 69372.
[3]McGill University, The Montreal General Hospital Research Institute, 2165, Cedar Avenue, Montreal PQH3G 1A4, Canada.
Received 5 September 1990; and in revised form 14 January 1991.

Cases considered to have a hereditary basis include: (1) all bilateral retinoblastomas and Wilms' tumours; (2) all children for which an established underlying genetic condition had been recorded; and (3) all tumours occurring in children with aniridia or hemihypertrophy. Excluded were cases with a history of a congenital malformation not specifically known to be associated with a defined genetic syndrome.

Information about family history was not routinely available, but was provided for some tumour types, including medullary carcinoma of the thyroid. Genetic cancer syndromes diagnosed on the basis of multiple primary tumours or the appearance of a subsequent benign manifestation of the syndrome will often be missed in the present analysis.

The relative risk for developing cancer was calculated by dividing the observed number of hereditary cancer cases by the expected number. The expected number was the product of the prevalence of the genetic condition taken from published reports and the total number of tumour registrations. The (two-sided) $P$-values and confidence limits associated with risk estimates were calculated assuming a Poisson distribution of the observed number of cases.

### Results

Of the total number of tumours reported, 509 or 3.07% had an underlying genetic condition recorded, the frequencies of which appear in Table I. The proportions of particular tumour types with genetic diagnoses are presented in Table II.

Bilateral retinoblastoma accounted for one-third of the genetic total; the median age at diagnosis of 162 bilateral tumours was 7 months, as compared to 25 months for unilateral tumours ($P<0.001$).

Down syndrome was the second most frequently cited condition. Of the 131 associated leukaemias 73 were acute lymphocytic, 49 were acute non-lymphocytic, one was chronic myelocytic and eight were classified as other, or unspecified leukaemia. A higher proportion of cases of acute non-lymphocytic leukaemia (5.3%) were attributable to Down syndrome than were acute lymphocytic leukaemias (1.7%). The relative risk for acute lymphocytic leukaemia was constant at different ages, in contrast to the acute non-lymphocytic subtype, where the greatest risk was seen before age 5 (Figure 1). There were three lymphomas associated with Down syndrome, including one in a phenotypic female with a 47,XY + 21/46,XO + 21 karyotype and testicular feminisation. One testicular teratoma was seen in a 14-year old boy. Other cancers, including two gliomas and one fibrosarcoma, were not seen more often than expected and have not been included in the heritable fraction.

**Table I** Genetic conditions listed in National Registry for Childhood Tumours

| | *No. of entries:* |
|---|---|
| Bilateral retinoblastoma | 162 |
| Down syndrome | 135 |
| Neurofibromatosis | 90 |
| Wilms' tumour[a] | 71 |
| Tuberous sclerosis | 20 |
| Ataxia-telangiectasia | 7 |
| Multiple endocrine neoplasia | 6 |
| Wiskott-Aldrich syndrome | 3 |
| Beckwith-Wiedemann syndrome[b] | 3 |
| Fanconi anaemia | 2 |
| 46XY gonadal dysgenesis | 2 |
| Turcot syndrome | 2 |
| Sturge-Weber syndrome | 1 |
| Bloom syndrome | 1 |
| Xeroderma pigmentosum | 1 |
| Hypogammaglobulinemia | 1 |
| IgA deficiency | 1 |
| Severe combined immunodeficiency | 1 |
| Total | 509 |

[a]Included in hereditary forms of Wilms' tumour are bilateral cases and cases associated with aniridia or hemihypertrophy. [b]These cases include one thyroid carcinoma, one non-Hodgkin's lymphoma and one hepatoblastoma and are distinct from cases associated with Wilms' tumour.

**Table II** Total number of cancers and proportion with genetic conditions

| | *Number of Children* | *Number with genetic conditions* | *%* |
|---|---|---|---|
| Leukaemias | 5,564 | 142 | 2.6 |
| Lymphomas | 1,781 | 17 | 1.0 |
| Brain and spinal cord | 3,872 | 79 | 2.0 |
| Sympathetic Nervous system | 985 | 2 | 0.2 |
| Retinoblastoma | 436 | 162 | 37.2 |
| Kidney | 984 | 71 | 7.2 |
| Liver | 135 | 3 | 2.2 |
| Bone | 850 | 0 | 0.0 |
| Soft tissue sarcoma | 1,003 | 20 | 2.0 |
| Gonadal and germ cell | 430 | 3 | 0.7 |
| Epithelial | 524 | 10 | 1.9 |
| Total | 16,564 | 509 | 3.1 |

Genetic conditions listed are those that appear in Table I.

**Figure 1** Risk of acute leukaemia associated with Down syndrome. White bars represent acute non-lymphocytic leukaemia and black bars represent acute lymphocytic leukaemia. Expected numbers based on an incidence of Down syndrome of 1.15 per 1000 (Neilsen & Sillesen, 1975).

Neurofibromatosis was found in excess in children with both acute lymphocytic and chronic myeloid leukaemia (Table III). Both cases of Hodgkin's disease were of the nodular sclerosis subtype. Sixty of 3872 (1.5%) children with tumours of the brain and spinal cord had neurofibromatosis, including 23.4% of malignant optic gliomas and 4.9% of meningiomas. Two tumours of the sympathetic nervous system, including one of the four recorded cases of malignant pheochromocytoma, were seen with neurofibromatosis. Neurofibromatosis was recorded in 12 of 16 cases of neurofibrosarcoma. For all cancers combined, the relative risk associated with neurofibromatosis was 16.3 (95% CI, 13.1 to 20.0). There were an additional 24 patients registered for whom only café au lait spots were recorded, including 15 with astrocytomas and five with other brain neoplasms. These have not been included in the genetic total.

Tuberous sclerosis is estimated to affect one in 15,000 children in Great Britain (Hunt & Lindenbaum, 1987). The relative risk of 18.1 for all types of cancer in these children (95% CI, 11.1 to 28.0) could be accounted for by a 70-fold increase in brain tumours and 50-fold risk for rhabdomyosarcoma (Table IV). The two rhabdomyosarcomas involved the cervix of a 12 year old female, and the kidney of a 14 year old male.

Ninety-eight per cent of kidney neoplasms were Wilms' tumours, and of these, 51 or 5.3% were bilateral. Bilateral Wilms' appeared earlier than the unilateral form (median age at diagnosis 19 months *vs* 38 months, $P<0.01$). The majority (82%) of bilateral Wilms' tumours presented synchro-

**Table III** Cancers associated with neurofibromatosis

| | Observed | Expected | Relative risk |
|---|---|---|---|
| All leukaemia | 7 | 1.85 | 3.8[a] |
| Acute lymphocytic | 4 | 1.46 | 2.7 |
| Chronic myelocytic leukaemia | 3 | 0.04 | 71.4[b] |
| All lymphoma | 3 | 0.59 | 5.1[a] |
| Hodgkin's disease | 2 | 0.25 | 8.0[a] |
| Non-Hodgkin's lymphoma | 1 | 0.30 | 3.4 |
| All brain and spinal cord | 60 | 1.29 | 46.5[b] |
| Ependymoma | 1 | 0.15 | 6.6 |
| Optic glioma | 34 | 0.05 | 920[b] |
| Other astrocytoma | 13 | 0.42 | 31.1[b] |
| Medulloblastoma | 3 | 0.26 | 11.4[a] |
| Meningioma | 3 | 0.02 | 155[b] |
| Other glioma | 6 | 0.21 | 29.0[b] |
| All sympathetic | 2 | 0.33 | 6.1[a] |
| Neuroblastoma | 1 | 0.32 | 3.1 |
| Pheochromocytoma | 1 | 0.001 | 1000[a] |
| All soft tissue sarcoma | 18 | 0.33 | 53.8[b] |
| Rhabdomyosarcoma | 5 | 0.21 | 23.4[b] |
| Neurofibrosarcoma | 12 | 0.005 | 9000[b] |
| Other fibrosarcoma | 1 | 0.05 | 20 |
| All cancers | 90 | 5.52 | 16.3[b] |

Expected numbers of tumours based on an incidence of neurofibromatosis of 1:3000 individuals (Crowe *et al.*, 1956). Because of the high proportions of optic gliomas, pheochromocytoma, meningiomas and neurofibrosarcomas associated with neurofibromatosis the odds ratio is used to approximate the relative risk. [a]$P<0.05$; [b]$P<0.001$

**Table IV** Cancers associated with tuberous sclerosis

| | Observed | Expected | Relative risk |
|---|---|---|---|
| Brain and spinal | 18 | 0.26 | 69.7[a] |
| Astrocytoma | 9 | 0.09 | 104[a] |
| Other glioma | 6 | 0.04 | 150[a] |
| Other CNS | 3 | 0.04 | 75[a] |
| Rhabdomyosarcoma | 2 | 0.04 | 50[a] |
| All cancers | 20 | 1.1 | 18.1[a] |

[a]$P<0.01$. Expected numbers of tumours based on an incidence of tuberous sclerosis of 1:15,000 individuals (Hunt & Lindenbaum, 1984).

nously; for the remaining nine bilateral tumours the interval between diagnoses ranged from 4 to 88 months. Twelve cases (1.2%) of Wilms' tumour occurred with aniridia (four with bilateral tumours, eight with unilateral). Hemihypertrophy appeared in two cases with aniridia and 12 additional cases without aniridia. Four of the 12 cases of Wilms' tumour associated with aniridia also had genital abnormalities. Isolated genitourinary abnormalities, although not considered sufficient evidence of genetic predisposition, were seen ten times more frequently among Wilms' tumour patients (2.0%) than among patients with other tumours (0.2%).

Primary liver cell tumours accounted for less than 1% of all cancers. Among 102 cases of hepatoblastoma there was one infant with the Beckwith-Wiedemann syndrome. Of the 33 cases of liver carcinoma, one was associated with hypogammaglobulinemia.

Less than 3% of the genetic cancers were attributable to immunodeficiency (Table V). The risk for lymphoma among children with ataxia-telangiectasia, based on a disease frequency of one in 100,000 (Pippard *et al.*, 1988) was 400 times greater than in the general population. One child with skin carcinoma and IgA deficiency was reported.

There were 11 cases of medullary thyroid carcinoma, representing 17.2% of childhood thyroid cancers. Six of these children were from families with multiple endocrine neoplasia type 2, four of whom had features of the mucosal neuroma syndrome.

Three other genetic conditions were included as underlying causes of carcinomas (which represent 3.5% of all childhood cancer): one child with hemihypertrophy and thyroid carcinoma; one with xeroderma pigmentosum and squamous cell carcinoma of the orbit; and one with Turcot syndrome and adenocarcinoma of the colon. In addition, malignant germ cell tumours were recorded in two phenotypic females with 46,XY gonadal dysgenesis.

### Discussion

An early age of onset is one of the features that discriminate between familial and sporadic cancer. We have estimated the hereditary fraction of childhood cancers retrospectively from information on genetic diagnoses in a large population-based series of cancer cases, an approach that does not require knowledge of gene frequencies and cancer penetrance. For some cancer types the numbers of cases are insufficient to estimate the effect of rare genetic traits with precision. The proportion of cancers are due to inherited mutations may be underestimated if documentation of constitutional abnormalities and other underlying conditions is incomplete. Registry cases which are genetic by virtue of either family history, chromosomal abnormalities or multiple primaries will have been overlooked in the above analysis; we attempt to estimate the size of these additional proportions from published reports in the following discussion.

Genetic forms of retinoblastoma other than bilateral include unilateral familial tumours, unilateral tumours in persons carrying 13q deletions and unilateral sporadic tumours associated with new germ cell mutations which are not visible cytogenetically. Tumours in the last category will not be considered to be familial at the time of diagnosis, but will be transmitted to 50% of the patient's children. Because the 13q deletion is rarely transmitted (Motegi *et al.*, 1983; Bunin *et al.*, 1989) the three subgroups of patients with unilateral tumours can be considered as non-overlapping. Of 123 unilateral cases in an American series, five were familial (4.1%) and a further six cases carried deletions of 13q (4.9%) (Bunin *et al.*, 1989). Applying these rates to the 274 unilateral cases in the Registry would yield 24.6 additional genetic cases. In an earlier report of retinoblastoma patients treated in Britain between 1950 and 1977, 44% of 882 children had either bilateral disease or a positive family history (Draper *et al.*, 1986). Forty per cent of a series of 598 French patients were hereditary by the same two criteria (Briard-Guillemot *et al.*, 1974). Retinoblastomas due to new mutations which are not detected cytogenetically may be ascertained through their offspring. Of 434 children of unilateral sporadic cases reported to 1979, 24 were affected (reviewed by Vogel, 1979), but none in a series of 94 more recently observed offspring developed retinoblastoma (Hawkins *et al.*, 1989). Assuming 90% penetrance, the combined recurrence risk of 4.5% implies a hereditary fraction of 10.1% for this subgroup (95% confidence interval, 6.7% to 15.1%). Adding 10% of the non-familial, unilateral cases (436−162 − 11.2 = 263 cases) to the total increases the genetic fraction of retinoblastoma

**Table V** Cancers associated with immunodeficiency states

| | Leukaemia n = 2 | Hodgkin's disease n = 2 | Non-Hodgkin's lymphoma n = 7 | Other n = 2 |
|---|---|---|---|---|
| Ataxia-telangiectasia | 0 | 2 | 5 | 0 |
| Severe combined immunodeficiency | 1 | 0 | 0 | 0 |
| Wiskott-Aldrich | 1 | 0 | 2 | 0 |
| Hypogammaglobulinemia | 0 | 0 | 0 | 1 |
| IgA deficiency | 0 | 0 | 0 | 1 |

to 49% (Table VI). This estimate is larger than the figure of 40% often quoted, and the 44% figure from the earlier Registry report (Draper *et al.*, 1986) but we have extended previous findings to include cases due to new mutations.

In the American National Wilms' Tumor Study, 37 of 3442 (1.1%) of children with Wilms' tumour had a positive family history. (Breslow *et al.*, 1988); 11% of all patients could be classified as genetic because of one or more of bilaterality (7.0% of the total); aniridia (0.8%) hemihypertrophy (3.3%) or family history. In a French series 13% of 298 patients were genetic by these criteria (Bonaitai-Pellié *et al.*, 1988). No recurrent cases were found among 179 (Li *et al.*, 1988) or 54 (Hawkins *et al.*, 1989) children of unilateral sporadic cases — a finding inconsistent with a hereditary fraction of greater than 3% for this subgroup ($P = 0.05$). The evidence for a constitutional mutation in patients with bilateral Wilms' tumour or with hemihypertrophy alone is less than for bilateral retinoblastoma because few offspring of these patients have been observed. Familial Wilms' tumour and Wilms' tumour with aniridia combined yield a much more conservative genetic proportion of 2.2%.

An analysis of 143 children with soft tissue sarcoma in the Manchester Children's Tumour Registry revealed 11 children with family histories suggestive of the Li-Fraumeni syndrome (Birch *et al.*, 1984). Two sarcomas occurred in siblings and six patients (one with a sibling with an adrenocortical tumour) had mothers with pre-menopausal or bilateral breast cancer. A further two patients had siblings with astrocytoma and one had a sibling with a Wilms' tumour. Of 73 patients with osteosarcoma in the Manchester Registry, six mothers had breast cancer, compared with 2.1 expected ($P < 0.05$) (Hartley *et al.*, 1986). Barring chance association, four of the tumours may be attributed to a variant of the Li-Fraumeni syndrome. These proportions need to be confirmed in other populations. Osteosarcomas also appear in families with hereditary retinoblastoma, either as second primary tumours or in individuals without prior disease. In a British study 6.0% of survivors of hereditary retinoblastoma had developed an osteosarcoma within 18 years of the original diagnosis (Draper *et al.*, 1986).

Childhood adrenocortical carcinomas appear in families at high risk for various neoplasms, including rhabdomyosarcoma, brain tumours, breast cancer, and osteosarcoma (Miller, 1978) and are often followed by tumours at other sites (Fraumeni, 1977; Levine 1978). Of thirty-three cases of childhood adrenocortical carcinoma in the Registry, three were known to have a first-degree relative with rhabdoymosarcoma (two siblings, one father) and two had second primary neoplasms recorded. One girl diagnosed with adrenocortical carcinoma at three months developed a breast sarcoma at

**Table VI** The estimated proportion of cancers with underlying genetic etiologies, incorporating family history

| *Condition* | *Total no. of cases with condition* | *Per cent* |
|---|---|---|
| Total leukaemia | 142 | 2.6 |
| Down syndrome | 131 | 2.4 |
| Neurofibromatosis | 7 | 0.1 |
| Deficiency | 2 | 0.0 |
| Others | 2 | 0.0 |
| Total lymphoma | 17 | 1.0 |
| Ataxia-telangiectasia | 2 | 0.4 |
| Neurofibromatosis | 3 | 0.2 |
| Wiskott-Aldrich syndrome | 2 | 0.1 |
| Others | 7 | 0.3 |
| Total brain and spinal cord | 79 | 2.0 |
| Neurofibromatosis | 60 | 1.5 |
| Tuberous sclerosis | 18 | 0.5 |
| Turcot syndrome | 1 | 0.0 |
| Total sympathetic | 2 | 0.2 |
| Neurofibromatosis | 2 | 0.2 |
| Total retinoblastoma | 212.9 | 48.8 |
| Bilateral retinoblastoma | 162 | 37.1 |
| Familial unilateral retinoblastoma | 11.2 | 2.5 |
| 13q deletions, unilateral | 13.4 | 3.1 |
| Sporadic unilateral retinoblastoma | 26.3 | 6.0 |
| Total kidney | 80.6 | 8.2 |
| Bilateral Wilms' | 51 | 5.2 |
| Unilateral Wilms' with aniridia or hemihypertrophy | 20 | 2.0 |
| Familial Wilms' | 9.6 | 1.0 |
| Total liver | 3 | 2.2 |
| Total bone | 44.7 | 5.7 |
| Li-Fraumeni syndrome | 38.5 | 4.5 |
| Hereditary retinoblastoma | 10.2 | 1.2 |
| Total soft tissue sarcoma | 97.2 | 9.7 |
| Li-Fraumeni syndrome | 77.2 | 7.7 |
| Tuberous sclerosis | 2 | 0.2 |
| Neurofibromatosis | 18 | 1.5 |
| Total gonadal and germ cell | 3 | 0.7 |
| 46XY gonadal dysgenesis | 2 | 0.4 |
| Down syndrome | 1 | 0.2 |
| Total epithelial | 15 | 2.9 |
| Multiple endocrine neoplasia type 2 | 6 | 1.1 |
| Li-Fraumeni syndrome | 5 | 1.0 |
| Others | 4 | 0.8 |
| All cancers | 696.4 | 4.2 |

Estimates with decimals incorporate information from published reports (see text). Integer estimates are based on National Childhood Tumour Registry data only. The tumours associated with the Li-Fraumeni syndrome in the epithelial category are adrenocortical carcinomas. No overlap is assumed between the three subgroups of unilateral retinoblastoma. The estimated number of bone tumours following retinoblastoma is based on the derived mean of the incidence at 12 years (3.6%) and at 18 years (6.0%) post-treatment, from Draper *et al.*, 1986.

age 14. In another child, an osteosarcoma developed 10 years after the initial diagnosis at age two. Two of these children have been reported previously (Hartley *et al.*, 1987). The two second primaries which developed among 17 survivors of one year or more represent a risk 167-fold greater than expected.

When the familial proportions of childhood cancers from the above series are incorporated, a more complete picture of the total hereditary pattern emerges (Table VI). We estimate that a total fo 4.2% of childhood cancer cases have a genetic basis; the largest contributions come from Down syndrome (0.8%) the Li-Fraumeni syndrome (0.7%) neurofibromatosis (0.5%) and tuberous sclerosis (0.1%). These probably represent minimum estimates of the fraction of cases due to single gene disorders because of under-reporting of genetic conditions by the contributing physicians. The proportions of children with a positive family history are also likely to be low in many published studies because of incomplete ascertainment and because cases are often reported before the siblings have completed the period of risk. On the other hand, reports from the literature may lead to over-estimates of the familial proportions if there is a tendency to selectively publish positive data.

For the other childhood tumour types the risk to relatives is small. Family aggregation in Hodgkin's disease may be due in part to shared HLA types (Hors *et al.*, 1985) and favours a multifactorial pattern of inheritance. Siblings of Hodgkin's disease patients have an increased risk 7-fold (Grufferman *et al.*, 1977) or greater (Hafez *et al.*, 1985*a*), but because of the rarity of Hodgkin's disease the size of the actual increase is small and risks are not specifically calculated for children. Acute leukaemia in siblings has also been documented (Miller, 1963; Draper *et al.*, 1977; Hafez *et al.*, 1985*b*), especially among younger cases.

Family clusters of neuroblastoma have been reported (Chatten & Voorhess, 1967; Pegelow *et al.*, 1975) but there is little information available from extensive patient series. Two familial cases were observed among 60 neuroblastoma cases treated at the M.D. Anderson Hospital, Houston (Knudson & Strong, 1972) and one of 246 children with neuroblastoma registered in Denmark from 1943–1980 had a positive family history (Carlsen, 1986). In other studies familial cases were not seen (Kramer *et al.*, 1987). The total hereditary fraction of neuroblastoma is probably less than 1%. None of a total of 48 children of apparently sporadic cases developed neuroblastoma in three follow-up series (Li & Jaffe 1974; Bundey & Evans 1982; Hawkins *et al.*, 1989).

Although family history is an established risk factor for adult testicular tumours (Dieckmann *et al.*, 1987), there is little evidence for a familial predisposition in children. In a recent review of 82 reported family occurrences the only childhood tumours were two seminomas in brothers aged 13 and 14 (Dieckmann *et al.*, 1987). Seminomas figured in 66 of the 82 affected first degree relative pairs, but this subtype represents only two per cent of childhood testicular cancer (Li & Fraumeni, 1972). More common childhood forms include embryonal carcinoma and teratocarcinoma (Exelby, 1980). In an American hospital-based series, none of 70 children with testicular tumours had a positive family history noted in the chart (Li & Fraumeni, 1972). None of the testicular tumours in the Registry were bilateral.

Malignant melanoma may cluster in families, and perhaps 10% of cases are the expression of the dysplastic nevus syndrome (Greene *et al.*, 1983). As younger cases are more likely to be familial, it is surprising that none of the 78 children reported by Bader *et al.* (1985) or 27 children presenting with malignant melanoma in St Judes Hospital, Boston had affected relatives (Pratt *et al.*, 1988). Information on associated conditions was seldom available for children with skin tumours in the Registry.

Excluding the bilateral tumours, Down syndrome and neurofibromatosis together account for three-fourths of the tumours in the Registry for which genetic syndromes are recorded. In a series of 5,406 children with acute childhood leukaemia entering clinical trials, 2.1% had Down syndrome — equivalent to a relative risk of 18.5 (Robison *et al.*, 1984). This figure is similar to the Registry figure (2.3%) and to others (Stewart *et al.*, 1958; Kardos *et al.*, 1983) but is greater than the 1.1% observed in surveys in Boston 1947–1965 (Fraumeni *et al.*, 1971) and Manchester 1954–1968 (Evans & Steward, 1972). The increase may reflect improved survival of children with Down syndrome, recently estimated to be 76.6% to age 15 in British Columbia (Baird & Sadovnick, 1989), as compared with 48% survival to age 3 at the time of the Manchester survey (Evans & Steward, 1972).

Although the finding of a single case of malignant testicular teratoma in a 14 year-old boy with Down syndrome is not statistically significant, it confirms other reports (Miller, 1970; Dexeus *et al.*, 1988; Baird & Sadovnick, 1988), including that of Mann *et al.* (1989) who found two children with Down syndrome among 61 cases of childhood testicular tumours when 0.07 cases were expected ($P<0.01$).

The malignant complications of neurofibromatosis are well known (Hope & Mulvihill, 1981). Of a total of 401 children hospitalised with neurofibromatosis in the five series summarised by Hope & Mulvihill (1981) and more recently (Blatt *et al.*, 1986) malignant tumours were identified in 7.2% – most commonly, brain (3.0%) neurofibrosarcoma (2.5%), and leukaemia (1.0%). An additional 10% of children had optic gliomas, but these include asymptomatic tumours discovered by routine screening. Although rhabdomyosarcoma was not reported in these series, five of 84 children with rhabdomyosarcoma reported by McKeen *et al.* (1978) and four of 115 children reported by Hartley *et al.* (1988) had a concomitant diagnosis of neurofibromatosis. Our estimate of 0.8% for rhabdomyosarcoma may reflect under-reporting, but for other sites in our series the documentation rate was high – our figure of 75% for neurofibrosarcoma agrees with the earlier estimates of Chabalko *et al.* (1974) and Storm *et al.* (1980). Higher attributable fractions than those seen in the Registry have also been reported by Merten *et al.* (1974) for childhood meningioma (23%) and by Hoyt & Baghdassarian (1969) for optic nerve gliomas (58%). Bader *et al.* (1980) evaluated 4,900 successive cases of childhood cancer for underlying genetic conditions and found that neurofibromatosis was mentioned in 0.8% of the total. We estimate the fraction of childhood cancer due to neurofibromatosis to be 0.5% which includes 1.8% of all soft tissue sarcomas and 1.5% of brain tumours.

Congenital immunodeficiencies which predispose to lymphoma and leukaemia in children include ataxia-telangiectasia, the Wiskott-Aldrich syndrome, the Chediak-Higashi syndrome, congenital agamaglobulinemia and the X-linked lymphoproliferative syndrome (Filipovich *et al.*, 1985; Purtilo *et al.*, 1975). Thirteen per cent of children with the recessive degenerative disease ataxia-telangiectasia, which has an incidence of one in 100,000 (Pippard *et al.*, 1988), will develop cancer by age 15 (60% of these are lymphomas and 27% leukaemia) (Morrel *et al.*, 1986). Fifty per cent of the cases of leukaemia reported to the Immunodeficiency Cancer Registry from 1973–1984 were associated with to ataxia-telangiectasia (Filipovich *et al.*, 1985) but ages of diagnosis were not given. Ataxia-telangiectasia was not associated with childhood leukaemia in the present study.

The reasons for the documented association of congenital defects and several cancers are not clear. In rare cases a prenatal exposure has been implicated (e.g. diethylstilbesterol, genital malformations and vaginal adenocarcinomas; Herbst *et al.*, 1975). In some, malformations and childhood neoplasms may be the various expressions of a single mutant gene, and in others, chromosomes may be deleted for contiguous genes with discrete effects. In the majority of children with Wilms' tumour and aniridia a deletion of 11p13 is detectable (Riccardi *et al.*, 1980). Other cases of Wilms' may be associated with hemihypertrophy, genitourinary abnormalities, (Breslow *et al.*, 1988) or cardiac septal defects (Stiller *et al.*, 1987) and may reflect abnormal embryonic development. Renal abnormalities have been documented in children with acute lymphoblastic leukaemia (Robison *et al.*, 1982) and hepatoblastoma is associated with hemihypertrophy (Fraumeni *et al.*, 1968) and familial polyposis (Kingston *et al.*, 1983; Li *et al.*, 1987). Defects of the neural tube, sacrum and pelvis are more common in children with germ cell tumours (Fraumeni *et al.*, 1973; Birch *et al.*, 1982).

If genetic counselling is to be applied to the prevention of cancer, it is first necessary to identify the individual at risk – but a positive family history will be seen in only a fraction of genetic cases. The vast majority of occurrences of Down syndrome are sporadic. Three quarters of patients with genetic retinoblastoma (Bunin *et al.*, 1989; Sanders *et al.*, 1988) and 89% of bilateral Wilms' tumour patients (Breslow *et al.*, 1988) occur in families with no other members affected. One-half of neurofibromatosis cases are the result of new mutations (Crowe *et al.*, 1956) and as average family size becomes smaller, the majority of new cases of recessive disease will be isolated as well.

The diagnosis of a few cancer syndromes is now possible by polymorphic DNA markers, including retinoblastoma (Wiggs *et al.*, 1988) and multiple endocrine neoplasia (type 2a (Sobol *et al.*, 1989)) or direct analysis of mutations (Yandell *et al.*, 1989) and early identification and surgery may benefit the individual at-risk. Prophylactic surgery is also advised for the rare female patient with gonadal dysgenesis who carries Y chromosome material. Although the recurrence risk in neuroblastoma has not been precisely established, screening of close relatives of cases by urinary catecholamines is probably justified.

The results of this survey and literature review suggest that roughly 4% of childhood cancers are directly attributable to genetic conditions. Because an underlying syndrome or a positive family history may be unrecognised or go unreported, this estimate is likely to be a minimum. Variations in incidence between ethnic groups for several tumour types and a lack of known environmental determinants suggest that the role of hereditary factors in childhood cancer may be considerably greater.

We are grateful to the many consultants and general practitioners who provided information on which this paper is based, and to the Office of Population Censuses and Surveys, the Information and Statistics Division of the Common Services Agency of the Scottish Health Service, the Registrar General for Scotland, the United Kingdom Children's Cancer Study Group and regional cancer registries for providing copies of notifications of childhood cancer cases. We thank Mr M. Loach, Mrs M. Allen and Dr E.L. Lennox for their work on the National Registry of Childhood Tumours. We are indebted to J.J. Mulvihill, G.J. Draper, J. Little, R. Montesano and L. Tomatis for helpful discussion. G. Bunin and C. Bonaitai-Pellié kindly provided information beyond that found in their published report.

The Childhood Cancer Research Group is supported by the Department of Health and the Scottish Home and Health Department. Data collection was also supported by the Marie Curie Memorial Foundation, the Cancer Research Campaign and the Medical Research Council. S. Narod was supported by the Ontario Ministry of Health and the Association pour la Recherche sur le Cancer.

### References

BADER, J.L., MEADOWS, A.T., LEMERLE, J. & 5 others (1980). Neurofibromatosis (NF) and other genetic defects associated with childhood cancer. *Am. J. Hum. Genet.*, **97A** (abstract).

BADER, J.L., STRICKMAN, N.A., LI, F.P., GREEN, D.M. & OLMSTEAD, P.M. (1985). Childhood malignant melanoma. Incidence and etiology. *Am. J. Pediatr. Hematol. Oncol.*, **7**, 341.

BAIRD, P.A. & SADOVNICK, A.D. (1988). Causes of death to age 30 in Down syndrome. *Am. J. Hum. Genet.*, **43**, 239.

BAIRD, P.A. & SADOVNICK, A.D. (1989). Life tables for Down syndrome. *Hum. Genet.*, **82**, 291.

BALE, S.J., DRACOPOLI, N.C., TUCKER, M.A. & 7 others (1989). Mapping the gene for hereditary cutaneous melanoma – dysplastic nevus to chromosome 1p. *N. Engl. J. Med.*, **230**, 1367.

BIRCH, J.M., MARSDEN, H.B. & SWINDELL, R. (1982). Pre-natal factors in the origin of germ cell tumors in childhood. *Carcinogenesis*, **3**, 75.

BIRCH, J.M., HARTLEY, A.L., MARSDEN, H.B., HARRIS, M. & SWINDELL, R. (1984). Excess risk of breast cancer in the mothers of children with soft tissue sarcomas. *Br. J. Cancer*, **49**, 325.

BIRCH, J.M. & MARSDEN, H.B. (1987). A classification scheme for chidhood cancer. *Int. J. Cancer*, **40**, 620.

BLATT, J., JAFFE, R., DEUTSCH, M. & ADKINS, J.C. (1986). Neurofibromatosis and childhood tumors. *Cancer*, **57**, 1225.

BONAITAI-PELLIE, C., CHOMPRET, A., TOURNADE, M.F., ZUCKER, J.M. & LEMERLE, J. (1988). Etude génétique et épidémiologique francaise sur le nephroblastome: resultats préliminaires. *Bull. Cancer*, **75**, 131.

BRESLOW, N.E., BECKWITH, J.B., CIOL, M. & SHARPLES, K. (1988). Age distribution of Wilms' tumor: report from the National Wilms' Tumor Study. *Cancer Res.*, **48**, 1653.

BRIARD-GUILLEMOT, M.L., BONAITAI-PELLIE, C., FEINGOLD, J. & FREZAL, J. (1974). Etude génétique du retinoblastome. *Humangenetik*, **24**, 271.

BUNIN, G.R., EMANUEL, B.S., MEADOWS, A.T., BUCKLEY, J.D., WOODS, W.G. & HAMMOND, G.D. (1989). Frequency of 13q abnormalities among 203 patients with retinoblastoma. *J. Natl Cancer Inst.*, **81**, 370.

BUNDEY, S. & EVANS, K. (1982). Survivors of neuroblastoma and ganglioneuroma and their families. *J. Med. Genet.*, **19**, 16.

CARLSEN, N.L.T. (1986). Epidemiological investigations on neuroblastomas in Denmark 1943–1980. *Br. J. Cancer*, **54**, 977.

CHABALKO, J.J., CREAGAN, E.T. & FRAUMENI, J.F. (1974). Epidemiology of selected sarcomas in children. *J. Natl Cancer Inst.*, **53**, 675.

CHATTEN, J. & VOORHESS, M.L. (1967). Familial neuroblastoma. Report of a kindered with multiple disorders, including neuroblastomas in four siblings. *N. Engl. J. Med.*, **277**, 1230.

CROWE, F.W., SCHULL, W.J. & NEEL, J.V. (1956). *A Clinical Pathological and Genetic Study of Multiple Neurofibromatosis.* Charles C. Thomas: Springfield.

DEXEUS, F.H., LOGOTHETIS, C.J., CHONG, C., SELLA, A. & OGDEN, S. (1988). Genetic abnormalities in men with germ cell tumors. *J. Urol.*, **140**, 80.

DIECKMANN, K.P., BECKER, T., JONAS, D. & BAUER, H.W. (1987). Inheritance and testicular cancer. Arguments based on a report of three cases and a review of the literature. *Oncology*, **44**, 367.

DOLL, R. & PETO, R. (1981). The causes of cancer. *J. Natl Cancer Inst.*, **66**, 1191.

DRAPER, G.J., HEAF, M.M. & KINNIER-WILSON, L.M. (1977). Occurrence of childhood cancers among sibs and estimation of family risks. *J. Med. Genet.*, **14**, 81.

DRAPER, G.J.., SANDERS, B.M. & KINGSTON, J.E. (1986). Second primary neoplasms in patients with retinoblastoma. *Br. J. Cancer*, **53**, 661.

DRAPER, G.J., STILLER, C.A., FEARNLEY, H., LENNOX, E.L., ROBERTS, E.M. & SANDERS, B.M. (1988). United Kingdom – England and Wales.. National registry of childhood tumours, 1971–1980. In Parkin, D.M., Stiller, C.A., Draper, G.J. *et al.*, (eds) p. 295. *International Incidence of Childhood Cancer.* IARC Scientific Publications: Lyon.

EXELBY, P.R. (1980). Testicular cancer in children. *Cancer*, **45**, 1803.

EVANS, D.I.K. & STEWARD, J.K. (1972). Down's syndrome and leukemia. *Lancet*, **ii**, 1322.

FILIPOVICH, A.H., ROBISON, L. & HEINITZ, K.J. (1985). Tumours in patients with naturally occurring immunodeficiency disorders: report from the Immunodeficiency Cancer Registry. In *Familial Cancer*, Muller, H. & Weber, J. (eds) p. 225. Karger: Basel.

FRANCOIS, J., MATTON, M.T., DEBIE, S., TANAKA, Y. & VANDERBULCKE, D. (1975). Genesis and genetics of retinoblastoma. *Ophthalmologica*, **70**, 405.

FRAUMENI, J.F., MILLER, R.W. & HILL, J.A. (1968). Primary carcinoma of the liver in childhood: an epidemiologic study. *J. Natl Cancer Inst.*, **40**, 1087.

FRAUMENI, J.F., ROSEN, P.J., HULL, E.W., BARTH, R.F., SHAPIRO, S.R. & O'CONNOR, J.F. (1969). Hepatoblastoma in infant sisters. *Cancer*, **24**, 1086.

FRAUMENI, J.F., MANNING, M.D. & MITUS, W.J. (1971). Acute childhood leukemia: epidemiologic study by cell type of 1,263 cases at the Children's Cancer Research Foundation in Boston, 1947–65. *J. Natl Cancer Inst.*, **46**, 461.

FRAUMENI, J.F., LI, F.P. & DELAGER, N. Teratomas in children: epidemiologic features. *J. Natl Cancer Inst.*, **51**, 1425.

FRAUMENI, J.F. (1977). Clinical patterns of familial cancer. In: *Genetics of Human Cancer*. Mulvihill, J.J., Miller, R.W. & Fraumeni, J.F. (eds) p. 223. Raven Press: New York.

GRUFFERMAN, S., COLE, P., SMITH, P.G. & LUKES, R.J. (1977). Hodgkin's disease in siblings. *N. Engl. J. Med.*, **296**, 248.

GREENE, M.H., GOLDIN, L.R., CLARK, W.H. & 6 others (1983). Familial cutaneous malignant melanoma: autosomal dominant trait possibly linked to the Rh locus. *Proc. Natl Acad. Sci.*, **80**, 6071.

HAFEZ, M., EL-TAHAN, H., EL-MORSI, Z. & 5 others (1985). Genetic susceptibility in Hodgkin's lymphoma. In *Familial Cancer*. Muller, H. & Weber, J. (eds) p. 175. Karger: Basel.

HAFEZ, M., EL-TAHAN, H., EL-MORSI, Z. & 4 others (1985). Genetic environmental interaction in acute lymphatic leukemia. In *Familial Cancer*. Muller, H. & Weber, J. (eds) p. 161. Karger: Basel.

HARTLEY, A.L., BIRCH, J.M., MARSDEN, H.B. & HARRIS, M. (1986). Breast cancer risk in mothers of children with osteosarcoma and chondrosarcoma. *Br. J. Cancer*, **54**, 819.

HARTLEY, A.L., BIRCH, J.M., MARSDEN, H.B., REID, H., HARRIS, M. & BLAIR, V. (1987). Adrenal cortical tumours: epidemiological and familial aspects. *Arch. Dis. Child.*, **62**, 683.

HARTLEY, A.L., BIRCH, J.M. & MARSDEN, H.B. (1988). Neurofibromatosis in children with soft tissue sarcoma. *Pediatr. Hematol. Oncol.*, **5**, 7.

HAWKINS, M.M., DRAPER, G.J. & SMITH, R.A. (1989). Cancer among 1,348 offspring of survivors of childhood cancer. *Int. J. Cancer*, **43**, 975.

HERBST, A.L., POSKANZER, D.C., ROBBOY, S.J., FRIEDLANDER, L. & SCULLY, R.E. (1975). Prenatal exposure to stilbesterol. *N. Engl. J. Med.*, **292**, 334.

HOPE, D.G. & MULVIHILL, J.J. (1981). Malignancy in neurofibromatosis. *Adv Neurol.*, **29**, 33.

HORS, J., GONY, J., ANDRIEU, J.M. & 6 others (1985). Participation of the major histocompatibility complex in the determination of familial malignancies. In *Familial Cancer*. Muller, H. & Weber, J. (eds) p. 213. Karger: Basel.

HOYT, W.F. & BAGHDASSARIAN, S.A. (1969). Optic nerve glioma or childhood. Natural history and rationale for conservative management. *Br. J. Ophthalmol.*, **53**, 793.

HUNT, A. & LINDENBAUM, R.H. (1984). Tuberous sclerosis: a new estimate of prevalence within the Oxford region. *J. Med. Genet.*, **21**, 272.

KARDOS, G., REVESZ, T., BULIN, A., FEKETE, G., VARGHA, M. & SCHULER, D. (1983). Leukemia in children with Down's syndrome. *Oncology*, **40**, 280.

KINGSTON, J.E., HERBERT, A., DRAPER, G.J. & MANN, J.R. (1983). Association between hepatoblastoma and polyposis coli. *Arch. Dis. Child.*, **58**, 959.

KNUDSON, A.G. & STRONG, L.C. (1972). Mutations and cancer: neuroblastoma and pheochromocytoma. *Am. J. Hum. Genet.*, **24**, 514.

KNUDSON, A.G., STRONG, L.C. & ANDERSON, D.E. (1973). Heredity and cancer in man. In *Progress in Medical Genetics*, **9**, Steinberg, A.G. & Bearn, A.G. (eds) p. 113. Grune and Stratton: New York.

KRAMER, S., WARD, E., MEADOWS, A.T. & MALONE, K.E. (1987). Medical and drug risk factors associated with neuroblastoma: A case-control study. *J. Natl Cancer Inst.*, **78**, 797.

LEVINE, G.W. (1978). Adrenocortical carcinoma in two children with subsequent primary tumours. *Am. J. Dis. Child.*, **132**, 238.

LI, F.P. & FRAUMENI, J.F. (1972). Testicular cancers in children: epidemiologic characteristics. *J. Natl Cancer Inst.*, **48**, 1575.

LI, F.P. & JAFFE, N. (1974). Progeny of childhood cancer survivors. *Lancet*, **ii**, 707.

LI, F.P., THURBER, W.A., SEDDON, J. & HOLMES, G.E. (1987). Hepatoblastoma in families with polyposis coli. *J. Amer. Med. Assoc.*, **257**, 2475.

LI, F.P., WILLIAMS, W.R., GIMBRERE, K., FLAMANT, F., GREEN, D.M. & MEADOWS, A.T. (1988). Heritable fraction of unilateral Wilms' tumor. *Pediatrics*, **81**, 147.

MANN, J.R., PEARSON, D., BARRETT, A., BARNES, J.M. & WALLENDSZUS, K.R. (1989). Results of the United Kingdom Children's cancer study group's malignant germ cell tumour studies. *Cancer*, **63**, 1657.

MCKEEN, E.A., BODTHURA, J., MEADOWS, A.T., DOUGLASS, E.C. & MULVIHILL, J.J. (1978). Rhabdomyosarcoma complicating multiple neurofibromatosis. *J. Pediatr.*, **93**, 992.